# Beyond apical ballooning: computational modelling reveals morphological features of Takotsubo cardiomyopathy

**DOI:** 10.1080/10255842.2019.1632836

**Published:** 2019-07-03

**Authors:** Giulia Pontecorboli, Giovanni Biglino, Elena Giulia Milano, Froso Sophocleous, Benedetta Biffi, Amardeep Ghosh Dastidar, Silvia Schievano, Carlo Di Mario, Chiara Bucciarelli-Ducci

**Affiliations:** a Bristol Heart Institute, University Hospitals Bristol NHS Trust, Bristol, UK;; b Cardiovascular and Thoracic Department, Careggi University Hospital, Florence, Italy; Department of Experimental and Clinical Biomedical Sciences, University of Florence, Florence, Italy;; c Translational Health Sciences, Bristol Medical School, University of Bristol, Bristol, UK;; d Department of Medicine, Section of Cardiology, University of Verona, Verona, Italy;; e Institute of Cardiovascular Science, University College London, London, UK

**Keywords:** Takotsubo cardiomyopathy, cardiac magnetic resonance, statistical shape modelling, computational modelling, myocardial deformation

## Abstract

Takotsubo cardiomyopathy (TCM) is characterized by transient myocardial dysfunction, typically at the left ventricular (LV) apex. Its pathophysiology and recovery mechanisms remain unknown. We investigated LV morphology and deformation in n = 28 TCM patients. Patients with MRI within 5 days from admission (“early TCM”) showed reduced LVEF and higher ventricular volumes, but no differences in ECG, global strains or myocardial oedema. Statistical shape modelling described LV size (Mode 1), apical sphericity (Mode 2) and height (Mode 3). Significant differences in Mode 1 suggest that “early TCM” LV remodeling is mainly influenced by a change in ventricular size rather than apical sphericity.

## Introduction

Takotsubo cardiomyopathy (TCM) is a defined type of cardiomyopathy, frequently precipitated by a stressful event and characterized by transient regional myocardial dysfunction. The latter is typically localized at the left ventricular (LV) apex and referred to as “apical ballooning” (Prasad et al. 2008).

The LV dysfunction in Takotsubo is known to be transient and subject to functional recovery within few months of the acute event. However, previous studies have highlighted that the rapid improvement in LV ejection fraction is not accompanied by a recovery of LV deformation, which continues to impaired at 4-month follow-up and associated with altered cardiac energetic status and persistence of symptoms (Schwarz et al. 2017; Dawson et al. 2015). Underlying mechanisms and features of the acute and recovery phase that could predispose to heart failure remain to be clarified (Dawson 2018).

Our study aimed to investigate ventricular morphology, function and deformation in TCM patients in the early stage of the disease using 2 D-Feature-tracking cardiac MRI (FT-CMR) and 3 D-Statistical Shape Modelling analysis (SSM).

## Materials and methods

Consecutive patients with definite diagnosis of TCM according to European Society of Cardiology criteria undergoing CMR at our Centre, were retrospectively selected and assigned to two groups according to the timing of CMR from the admission: “early TCM” when CMR was performed by the 5^th^ day of hospitalization and “late TCM” when performed from the 5^th^ to the 11^th^ day from the admission.

**Figure 1.**
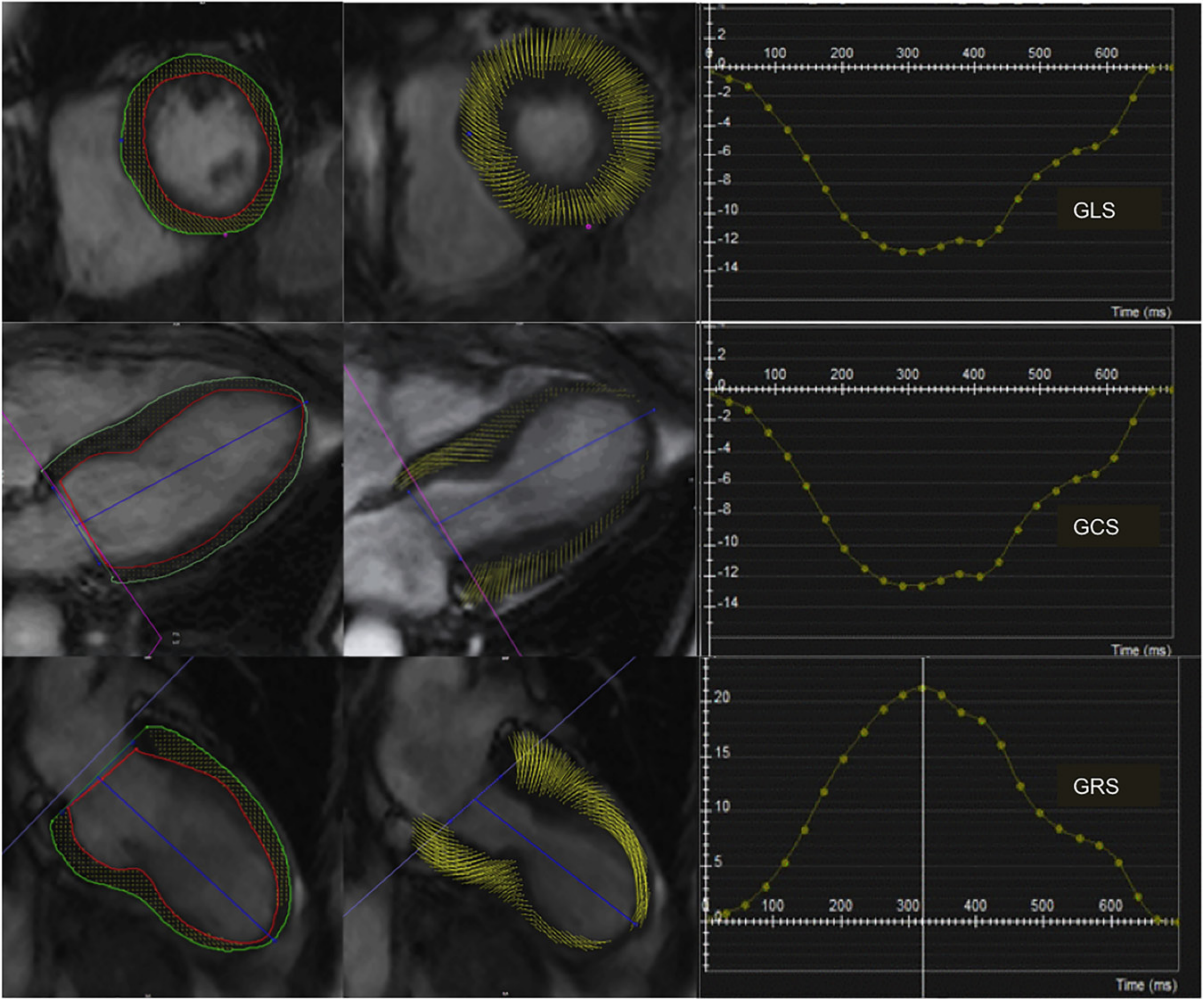
CMR-feature tracking in Takotsubo Cardiomyopathy: Global Longitudinal Strain (GLS) and Global Circumferential Strain (GCS) and Global Radial Strain (GRS), are calculated from the short axis, four-chamber and two-chamber SSFP cine images (cvi42, Circle Cardiovascular Imaging).

Patients with evidence of late gadolinium enhancement (LGE) on CMR imaging or with history of cardiac disease were excluded. 12-Lead ECG acquired ±48 hours from CMR were collected and analyzed. Myocardial edema was identified and quantified by semi-quantitative analysis on T2-STIR images (SI ratio of myocardium over skeletal muscle of ≥2.0). Global longitudinal, radial and circumferential strains were calculated with FT-CMR analysis (Figure 1). CMR cine images in short-axis view provided the input data for the statistical shape modelling (SSM) framework. Images were segmented creating 3 D volume meshes of LV endocardium at end-systole (ES) and end-diastole (ED) (Segment v2.0, Medviso, Lund, Sweden). A mean systolic LV configuration (‘template’) and deformations were computed, extracting shape vectors for further statistical analyses (Bruse et al. 2016). Principal component analysis (PCA) was applied to the set of computed deformation vectors in order to reduce the complex 3 D shape variation to only few components, or “shape modes”, recapitulating dominant shape features in this population. The analysis focused on ES modes to describe the apical ballooning.

## Results

**Figure 2.**
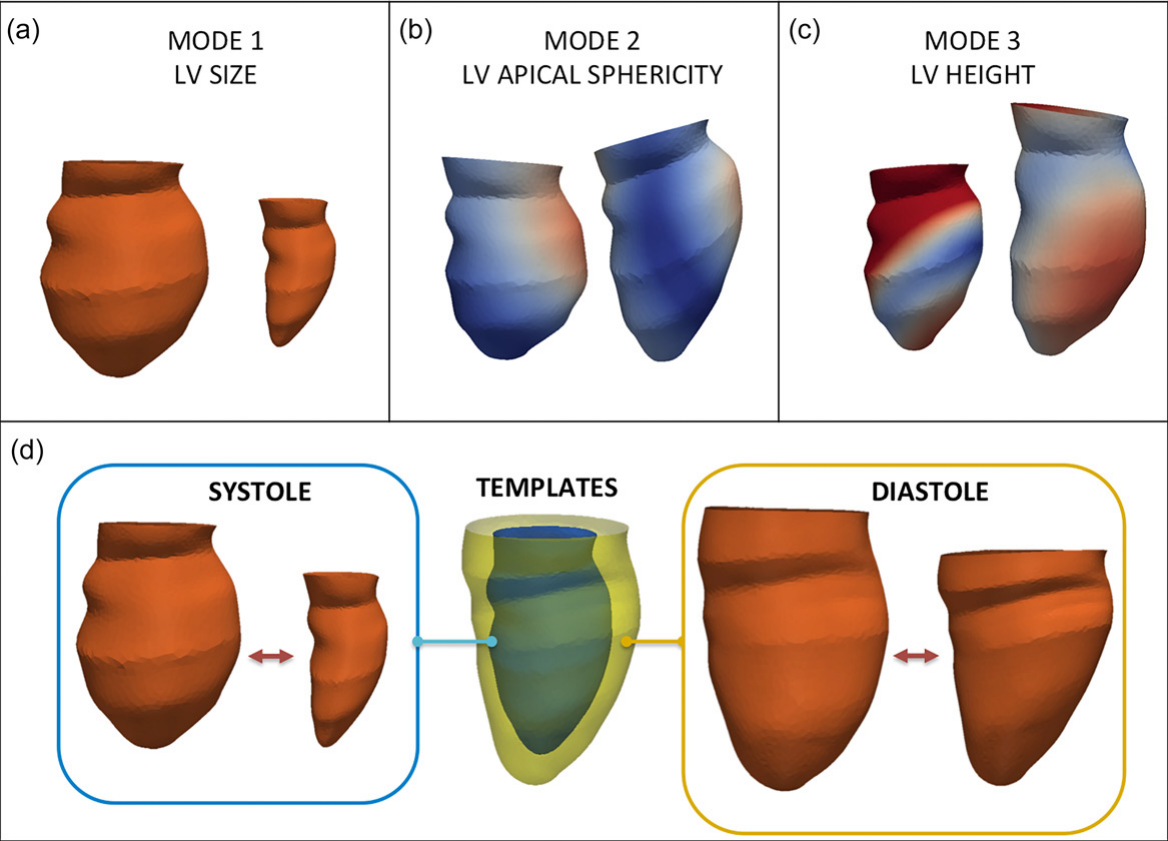
Shape modes derived from statistical shape modelling describe left ventricular (LV) size (a), apical sphericity (b) and height (c). The templates (d) show the mean configuration in the whole population at end-systole and end-diastole, recapitulating the variability in the population, as shown in the extreme systolic (blue) and diastolic (yellow) configurations.

Twenty-eight patients were included in the study (96% female, average age 65 ± 10 years), with equal distribution in the “early TCM” and “late TCM” sub-groups (14 patients each). Patients of the “early TCM” cohort exhibited lower LVEF (49 ± 11% vs. 60 ± 11%, p = 0.01), higher end-diastolic volumes (EDV, 82 ± 16 vs. 70 ± 21 mL/m^2^, p = 0.02) and end-systolic volumes (ESV, 42 ± 15 vs. 29 ± 17 mL/m^2^, 0.01) compared with “late TCM” group. No significant differences between the two cohorts were found in ECG parameters (heart rate, QTc interval, QT dispersion, number of leads with inverted T-waves, maximum depth of T-wave inversion), nor in the CMR-derived amount of edema (% LV mass and grams), nor in 2 D-FT CMR global strains. For SSM, two patients were excluded due to sub-optimal image quality. For the remaining 26 patients, the ES and ED templates were computed (Figure 2). The dominant shape mode (Mode 1), recapitulating almost 50% of the overall shape variability in the population and reflective of LV size, differed significantly between the two cohorts both in ES and in ED, showing increased values in the “early TCM” group, and correlated significantly with all three global strain components (p ≤ 0.005). Interestingly, Mode 2 depicted LV apical sphericity and Mode 3 depicted LV height but they did not differ significantly in the two cohorts.

## Discussion

Our study investigated ventricular morphology and deformation by means of 2 D-FT-CMR and 3 D-SSM in patients with TCM in acute and sub-acute phases. According to 2 D-CMR data, LVEF was lower in the “early TCM” cohort and both EDV and ESV were larger, yet no difference in the amount of myocardial edema and strain values was found. This is the first time that a statistical shape modelling framework is applied to elucidate 3 D-LV morphology in the presence of TCM, recapitulating in a single parameter the traditional Japanese octopus pot (tako-tsubo). The dominant shape mode, reflective of LV size, was significantly different in the two cohorts, and the results of our study suggest that the ventricular remodeling during the early stage of TCM is mainly influenced by an acute global dilatation of the LV (LV expansion), and less related to the sphericity of the apex.

Although our study is limited by its retrospective nature and the small population, it shows that SSM holds promise to explore nuances in morphological variability in TCM and that it could contribute to identify potential shape biomarkers to improve patients’ risk stratification.
